# Research on the construction of weaponry indicator system and intelligent evaluation methods

**DOI:** 10.1038/s41598-023-46660-5

**Published:** 2023-11-08

**Authors:** Shuai Wang, Yuhong Du, Shuaijie Zhao, Jinhu Hao, Lian Gan

**Affiliations:** 1 School of Mechanical Engineering, Tiangong University, 399 Bin Shui West Road, Xiqing District, Tianjin, 300387 China; 2 Key Laboratory of Advanced Mechatronics Equipment Technology, Tiangong University, 399 Bin Shui West Road, Tianjin, 300387 China

**Keywords:** Scientific data, Electrical and electronic engineering

## Abstract

To decrease subjective interference and improve the construction efficiency of the traditional weapon and equipment index system, an index system construction method based on target detection is proposed in combination with the equipment test video data. The three-level index system of combat effectiveness of a certain type of equipment is established, and various intelligent assessment methods are proposed. Firstly, an optimaized IPSO-BP network model is proposed, in which dynamic weights are set to improve the particle search network, and adaptive learning factors are introduced to optimize the update speed. Secondly, an improved DS evidence-parallel neural network assessment method is proposed, setting multiple parallel neural networks with different parameters, and improving the angle cosine to weaken the numerical nonlinear attributes in DS evidence fusion and increase the model's assessment operation stability. Thirdly, the three types of view features corresponding to the index item images are extracted to train the base classifiers. The integrated CNN network based multi-view feature integration assessment model is constructed and the improved residual network block is introduced to optimize the network gradient. Comparison with existing evaluation methods shows that the proposed methods achieve efficient and intelligent construction and evaluation of the indicator system and enrich the evaluation of indicator data.

## Introduction

As the functions of weapons and equipment become more refined and the structure becomes more complex, traditional indicator system construction methods may incorporate builders’ subjective will, resulting in incomplete and unscientific indicator systems. Compared with intelligent methods, the commonly used subjective and objective evaluation methods have lower efficiency and reliability in evaluation work. How to construct a scientific and comprehensive effectiveness index system for weapons and equipment, and apply intelligent evaluation methods to its efficient evaluation, is important in combat indicators research^1^.

Operational effectiveness is a measure of the effective role played by weapons and equipment in fulfilling operational tasks under certain conditions^2^. A reasonable combat effectiveness index system can provide effective guidance and guarantee for the research and development of new equipment, assessment of the health of in-service equipment, and the conduct of combat test activities^3^. The construction of weapon and equipment combat effectiveness index systems is usually based on theoretical research frameworks^4^, including combat missions^5^ and combat concepts^6,7^, or supplemented by research methods like analytical simulation and data modeling for optimization^8–10^. The above methods focus on the equipment’s tactical technical performance requirements, while the indicators’ dimensionality reduction process of which is tedious and limited by the subjectivity of expert experience. Image recognition technology is applied to index system construction to improve objectivity and accuracy in many fields. Fan et al.^11^ constructed a fire risk index system for industrial buildings by developing image recognition software to process fire-prone items. Zhang et al.^12^ and Sun et al.^13^ established multi-source image databases and optimized the index system using image data. The current troops have a large number of combat test video data, and there have been studies on index systems construction through image data classification, which provides a feasible reference for the construction of effectiveness index systems of weapons and equipment based on image recognition^14^.

The assessment methods of weapon and equipment effectiveness index system can be divided into subjective, objective, and intelligent methods^15^. The first class of methods, including fuzzy hierarchical analysis^16^ and cloud modeling^17^, is mainly based on questionnaires and expert consultation, where the assessment difficulty increases with the complexity. Data-driven objective assessment models are usually based on structural equation modeling(SEM)^18^, availability dependability capability modeling (ADC)^19^, weapon system of systems model (WSoS)^20^, and Bayesian network model (BN)^21^. These methods were developed to improve model credibility and computational power but failed to perform a deep and systematic analysis of indicators. The intelligent evaluation method introduces neural networks and integration strategies into the indicator evaluation process and lays a good foundation for exploring new intelligent assessment methods. Firstly, a multi-level long-term and short-term memory network^22^ can be constructed to characterize the functional mapping relationship between group structure, combat effectiveness, and individual decision-making. Secondly, the assessment model with a fully connected deep regression network, selecting fewer hidden layers and increasing the training volume, can achieve multi-indicator performance search^23,24^. Thirdly, the multi-attribute group decision-making method can be used to convert heterogeneous opinions into random values, and balance and rank these values for the selection and evaluation of missile weapon systems^25^.In addition, the strategy fusion method can be used to connect different single indicator processing models and increase the indicator system evaluation’s generalization performance^26^. The knowledge-model-based simulation system also provides a good foundation for exploring new automated intelligent assessment methods^27^.

Based on the combat test video data of the Army’s certain type of equipment, this paper selects 3 types of effects, 10 capability elements, and 29 index items that affect combat effectiveness. The combat effectiveness index system construction method of weapons and equipment based on target detection is proposed. There are 22 index items retained to construct the combat effectiveness index system based on corresponding typical detected objects’ recognition rates in ten test scenarios. We propose intelligent evaluation algorithms including “Optimised IPSO-BP neural network method”, “Improved DS evidence-parallel neural network method”, and “Multi-view feature based integrated residual network method” to increase the recognition accuracy and recall rate. Compared with different assessment methods, the three intelligent assessment methods realize a fully intelligent process from the input of indicator data to the output of assessment results, improving the evaluation reliability, rationality, and efficiency simultaneously.

## Research method

### Indicator system construction based on target detection

According to the research of the U.S. Army’s “Test and Certification Management Guide”^28^ and Shi et al.^29^ on the definition and classification of the factors affecting the combat effectiveness of weapons and equipment, the factors affecting combat effectiveness are divided into three typical categories—firepower application, co-operation, and command and control factors. Furthermore, it is divided into 10 capability factors, such as rapid response capability, in-vehicle cooperation capability, and situational awareness capability, in the process of testing weapons and equipment’s combat effectiveness in different environments. A total of 29 indicators, such as search range and combat readiness, are selected based on continuous/discrete and other indicator types, and typical detected objects corresponding to each indicator item are identified. Referring to Tian et al.^30^ definition of air combat control effectiveness index system’s gaze time index as the duration from the target discovery time sampling point to the target acquisition point, the search range index in this paper is defined as the moving trajectory of the aiming frame for the hitting target in the visual field. This index’s typical detection object is the aiming frame. The combat readiness time indicator is determined to identify the operation panel start heating switch button corresponding to the indicator light from bright to dark state, and the typical detection object is the indicator light state.

**Figure 1:**
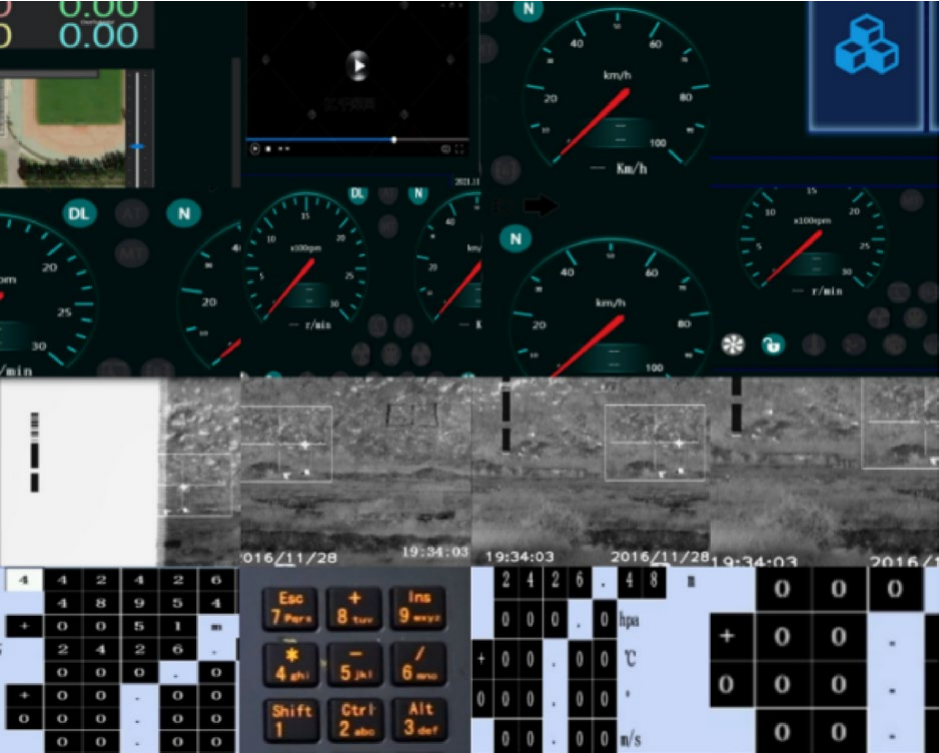
Data set of typical identified objects.

Based on the object representations of quantitative responses such as time and distance and the type representations of qualitative responses such as adaptability and condition, the typical indicator images and video data to be collected for the effectiveness indicator system construction are determined. The original image and video were collected from the driver operation terminal, artillery commander task terminal, relevant operation console panel, and simulation experimental platform of a certain army ground weapon equipment during combat test, with a total of 5924 images. To ensure the training of the image recognition network model and intelligent and optimal recognition, each image in the sample dataset contained one or more typical recognition objects. We labeled the original images based on 29 typical recognition objects, with the label named Key Indicators using the LabeIMe tool. Images that do not contain key indicator items were screened out, forming the corresponding equipment’s combat effectiveness test indicator image dataset, with a total of 4377 images. The training set, test set, and validation set were divided into 7:2:1. The constructed partial sample set is shown in Fig. 1. The weapon and equipment index systems should not only have clear definitions and meanings of indicators but also emphasize the repeatability in multiple typical combat environments^31^. Therefore, 10 test experiment scenarios were set up in the test trials. They are A: target search time; B: start-up heating time; C: network connectivity rate; D: intelligence formulation efficiency; E: NBC response time; F: firing reaction time; G: continuous firing speed; H: average mobile marching speed; I: target indication accuracy; and J: anti-interference capability.

### Optimizing IPSO-BP neural networks

Chen et al.^32^ found that using BP neural network to evaluate the index system may cause the “local minimum” problem by setting the network weights and thresholds in the operation process. The running time is longer for the reason that the error is back-propagated in the network. The PSO algorithm is hereby introduced to continuously seek the optimal solution. We propose to improve the dynamic IPSO-BP neural network model, optimize the initial weights and thresholds, and dynamically adjust the weight ratios of the two algorithms in each generation of the model, to achieve the index system intelligent assessment.

The weights and thresholds in the BP gradient descent network corresponding to the global optimal particles are introduced in the particle velocity search process, the output error value of the BP neural network is used as the suitability function, and the value of the BP neural network with the optimal suitability is imported into the particle velocity calculation. Dynamic coefficient $\varepsilon$ is set up to adjust the network occupancy ratio of the two algorithms of IPSO and BP gradient descent. The ratio of the current weight change to the last weight change is adjusted in each generation of weight update, to achieve global numerical optimization after many iterations. The improved particle search formula is as Eq. (1):

1 $$\left\{ {\begin{array}{*{20}l} {V_{mn}^{l + 1} = \left( {1 - \varepsilon } \right)\left[ {\begin{array}{*{20}c} {V_{mn}^{l} + c_{1} r_{1} \left( {P_{mn}^{l} - X_{mn}^{l} } \right)} \\ { + c_{2} r_{2} \left( {P_{gn}^{l} - X_{mn}^{l} } \right)} \\ \end{array} } \right] + \varepsilon V_{BP} } \hfill \\ {X_{mn}^{l + 1} = X_{mn}^{l} + V_{mn}^{l + 1} } \hfill \\ {\varepsilon = \frac{l}{2L}} \hfill \\ \end{array} } \right.$$

From Eq. (1): *V* and *X* are the velocity and position of the particle respectively, *m* and *n* are the nth dimensions of the mth particle. *l* is the current number of iterations and a random number within $\left( {0,1} \right)$. $c_{1}$ and $c_{2}$ are the learning factors, $p_{mn}$ and $p_{an}$ are the individual extreme value and the overall optimal fitness values respectively. $V_{Bp}$ is the optimal fitness particle based on the BP network value.

The formula for the improved weight $\omega$ and fitness-containing *k* learning factor in terms of particle update speed is as Eq. (2).

2 $$\left\{ {\begin{array}{*{20}l} {\omega = \frac{{ - 2l\left( {\omega_{max} - \omega_{min} } \right)}}{L} + \frac{{\omega_{max} - \omega_{min} }}{{1 + e^{{ - \left( {\frac{10}{L}l - 5} \right)}} }} + \omega_{m} } \hfill \\ {c_{1} = 2 + \frac{{k - k_{ave} }}{{k_{ave} - k_{min} }}} \hfill \\ {c_{2} = 2 - \frac{{k - k_{ave} }}{{k_{ave} - k_{min} }}} \hfill \\ \end{array} } \right.$$

Log curve decay weights^33^ are introduced in Eq. (2). The inertia change of the weights is decomposed into an initial decline to improve the global search ability and the particles’ convergence efficiency; an increase in the middle period to expand the model’s spatial search ability and global optimization ability; and a further decline in the late period to increase the local optimization to obtain high-precision values.$\omega$ is the inertia weight, which embodies the influence of the current particle search speed on the evolved particles' speed, controlling the model search performance. $\omega_{max}$ and $\omega_{min}$ are the maximum and minimum inertia weights respectively. A nonlinear function with fitness *k* is introduced to adaptively change the dynamic learning factors^34^ $c_{1}$ and $c_{2}$ to increase the weights of the individual extremes in the early stage of the algorithm, and the weights of the whole extremes in the later stage. $k_{ave}$ and $k_{min}$ are the maximum and minimum inertia weights respectively.

**Figure 2:**
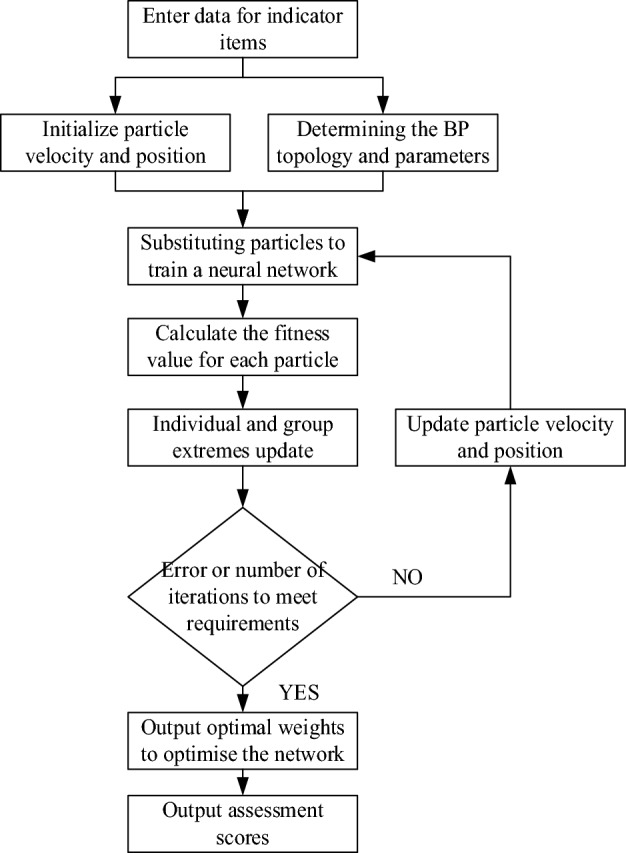
IPSO-BP model run flow chart.

The flow of the optimized IPSO-BP intelligent evaluation model is shown in Fig. 2. Constructing and normalizing the index data set. After initializing the particle’s velocity and position and determining the BP network’s parameters and structure, the IPSO algorithm calls the BP neural network to calculate the suitability value in the first iteration and calculate the particles’ suitability values in each generation to seek the suitability extremes of the particles individually and as a whole. According to Eqs. (1) and (2), the particle velocity and position are updated, and it is judged whether the maximum number of iterations or the preset error value is reached. If not, the particle velocity and position are continued to be updated using the gradient descent method for training. The BP suitability values will be called for weights and thresholds to find the optimal value, and finally, the model’s evaluation score can be obtained.

### Improving DS evidence-parallel networks

The statistical results of the optimized IPSO-BP model training data indicate that the model may generate significant evaluation errors for the poor stability of a single network. Therefore, a parallel neural network is proposed to improve the data processing.

$E_{i} = \left[ {B_{i1} ,B_{i2} , \ldots ,B_{iM} } \right]{ }$ is the output of the neural network $B_{i}$(*i* = 1,2,…,*A*),$B_{ij} { }$ is the jth node output of $B_{i}$, $E_{i}^{ + }$ = [$B_{i1}^{ + } ,B_{i2}^{ + } , \cdots ,B_{iM}^{ + }$] is the normalized result of $E_{i}$*, 1* $\le a \le M$, and the formula is as Eq. (3).

3 $$\left\{ {\begin{array}{*{20}l} {{\text{B}}_{{\text{i}}}^{ + } = \frac{{{\text{B}}_{{{\text{ij}}}}{^\prime} }}{{\mathop \sum \nolimits_{{{\text{a}} = 1}}^{{\text{M}}} {\text{B}}_{{{\text{ij}}}}{^\prime} }}} \hfill \\ {{\text{B}}_{{{\text{ij}}}}{^\prime} = \frac{{{\text{B}}_{{{\text{ij}}}} - {\text{min}}\left( {{\text{B}}_{{{\text{ia}}}} } \right)}}{{{\text{max}}\left( {{\text{B}}_{{{\text{ia}}}} } \right) - {\text{minB}}_{{{\text{ia}}}} }}} \hfill \\ \end{array} } \right.$$

If there exists ${ }B_{ig}^{ + } \in E_{i}^{ + }$ which satisfies all the assessment criteria, then there exists Eq. (4).4

After normalization and combining with the processing results of the test sample set, the formula for calculating the credibility $\theta_{i} \left( {\theta_{i} \in \left[ {0,1} \right]} \right)$ of the ${\text{B}}_{{\text{i}}}$ neural network is obtained in Eq. (5).

5 $$\theta_{i} = \frac{{\mathop \sum \nolimits_{l = 1}^{D} \frac{{\alpha_{1} }}{{\alpha_{1} + \beta_{1} }}}}{D} = \frac{{\mathop \sum \nolimits_{l = 1}^{D} \frac{{\alpha_{1} }}{{M_{l} - \gamma \alpha_{1} }}}}{D}$$

In Eqs. (6) and (7), $\varepsilon_{i} { }$ is the predetermined threshold, $M_{l}$ is the total number of test sample sets, $\alpha_{1}$ is the number of correct neural network evaluations, $\beta_{1}$ is the number of incorrect neural network evaluations, and $\gamma_{1}$ is the number of neural networks refusing to give results. The initial evaluation results are fused after the refinement of the DS evidence theory. Combining the credibility $\theta_{i} { }$ of the $B_{i}$(i = 1,2,…,A) neural network, a modified treatment of the preliminary evaluation results $K_{i}^{ + }$ is made from Eq. (6).

6 $${\text{p}}_{{\text{i}}} \left( {{\text{Q}}_{{\text{j}}} } \right) = \left\{ {\begin{array}{*{20}l} {{\uptheta }_{{\text{i}}} {\text{B}}_{{{\text{ij}}}}^{ + } ,} \hfill & {{\text{Q}}_{{\text{j}}} \ne \Theta } \hfill \\ { - \mathop \sum \limits_{{{\text{a}} = 1}}^{{\text{M}}} {\text{p}}_{{\text{i}}} \left( {{\text{Q}}_{{\text{a}}} } \right),} \hfill & {{\text{Q}}_{{\text{j}}} = {\Theta }} \hfill \\ \end{array} } \right.$$

The assignment of basic probability values for the generation of the Exhibit is $U_{i} = \left( {p_{i} \left( {Q_{1} } \right),p_{i} \left( {Q_{2} } \right), \ldots ,p_{i} \left( {Q_{M} } \right),p_{i} \left( \Theta \right)} \right)$. Let there be *m* mutually independent pieces of evidence $v_{i}$ in $\Theta$. Forming a matrix $X_{m \times N}$ based on the BPA of each piece of evidence as a row vector, where *N* is the number of propositions:

7 $$BetP_{{v_{i} }} (F_{j} ) = \mathop \sum \limits_{{F_{j} \subseteq F}} \left( {\frac{1}{\left| F \right|}} \right)v_{i} \left( F \right),\,\,F \in v_{i}$$

**Figure 3:**
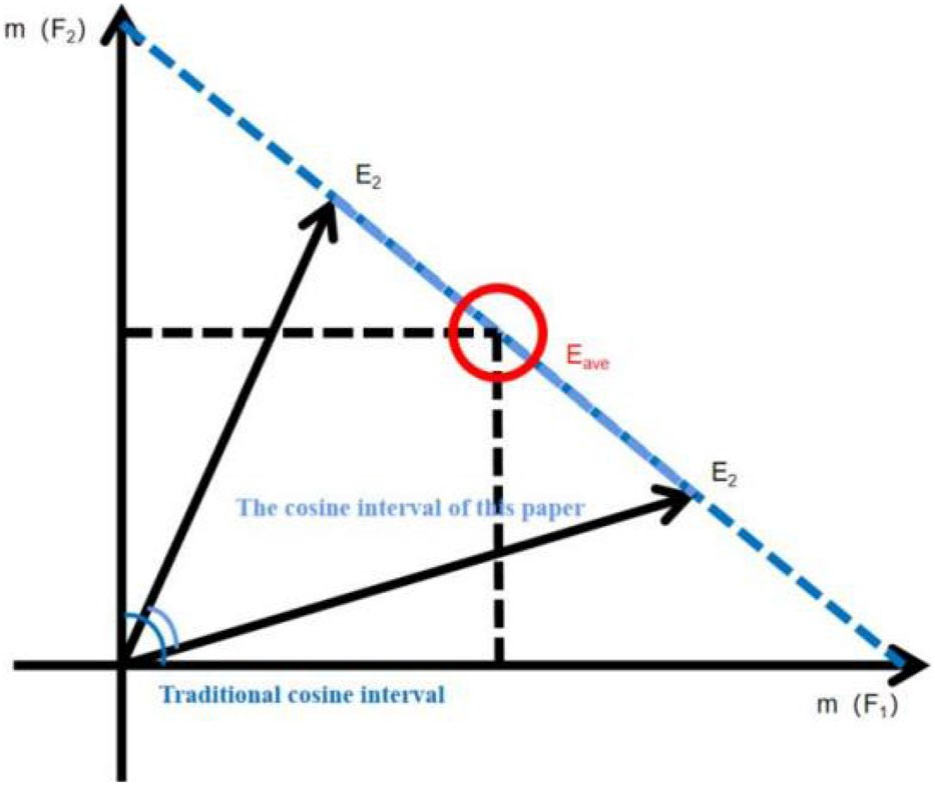
Improved cosine algorithm.

$BetP_{{v_{i} }} (F_{j} )$ in Eq. (7) is the Pig probability of ${ }F_{j} { }$ under the basic trust allocation $v_{i}$ , and *|F|* denotes the number of singletons in *F*. The BPA of multiple singleton propositions are equally allocated to each of them, and the evidence matrix $X_{m \times N}{^\prime}$ is collated by calculating the pig probabilities. Based on $p_{i} = \left( {v_{i} \left( {F_{1} } \right),v_{i} \left( {F_{2} } \right), \ldots v_{i} \left( {F_{m} } \right)} \right)$, let vector $p_{i} \left( {i = 1,2, \ldots ,m} \right)$ be the *i*th row of matrix *X*. The distribution of focal elements in the above evidence shows that all evidence exists in the first quadrant of the coordinate system, and all results are distributed in the interval [0,1] and non-linear if calculated directly using the cosine formula. The method of subtracting an average of all dimensions and then performing vector cosine calculation is used to make the results fall in the interval [− 1,1], which weakens the non-linear property of the results, as shown in Fig. 3.

Since there are* M* focal elements in $p_{i}$, the average value of the subtracted in Eq. (8).

8 $${\text{E}}_{{{\text{ave}}}} = {\text{m}}_{{{\text{ave}}}} \left( {{\text{F}}_{{\text{j}}} } \right) = \frac{1}{{\text{N}}}{ }\,\,\,{\text{j}} = 1,2, \ldots { },{\text{ N}}$$

where the $m_{ave}$ function is calculated by the number of evidence sources.

Then the improved cosine of the new evidence matrix $X_{m \times N}{\prime}$ , where exists vectors of $P_{\alpha }$, $P_{\beta }$, $\alpha$, $\beta \in i$, from Eq. (9).

9 $${\text{d}}_{\alpha \beta } = {\text{COS}}_{\alpha \beta } = \frac{{<{{\text{p}}_{\alpha } ,{\text{p}}_{\beta } }> }}{{| {{\text{p}}_{\alpha } |\cdot |{\text{p}}_{\beta } }|}} = \frac{{\mathop \sum \nolimits_{{{\text{l}} = 1}}^{{\text{M}}} {\text{p}}_{\alpha 1} {\text{p}}_{\beta 1} }}{{\sqrt {\mathop \sum \nolimits_{{{\text{l}} = 1}}^{{\text{M}}} \left( {{\text{p}}_{\alpha 1} } \right)^{2} } \sqrt {\mathop \sum \nolimits_{{{\text{l}} = 1}}^{{\text{M}}} \left( {{\text{p}}_{\beta 1} } \right)^{2} } }}$$

The cosine matrix is obtained as Eq. (10):

10 $${\varvec{E}}_{{{\varvec{m}} \times {\varvec{m}}}} = \left[ {\begin{array}{*{20}c} {\begin{array}{*{20}c} 1 & {e_{12} } & \cdots \\ {e_{21} } & 1 & \cdots \\ \vdots & \vdots & \ddots \\ \end{array} } & {\begin{array}{*{20}c} {e_{1\beta } } & \cdots & {e_{1m} } \\ {e_{2\beta } } & \cdots & {e_{2m} } \\ \vdots & {\mathinner{\mkern2mu\raise1pt\hbox{.}\mkern2mu \raise4pt\hbox{.}\mkern2mu\raise7pt\hbox{.}\mkern1mu}} & \vdots \\ \end{array} } \\ {\begin{array}{*{20}c} {e_{\alpha 1} } & {e_{\alpha 2} } & \cdots \\ \vdots & \vdots & {\mathinner{\mkern2mu\raise1pt\hbox{.}\mkern2mu \raise4pt\hbox{.}\mkern2mu\raise7pt\hbox{.}\mkern1mu}} \\ {e_{m1} } & {e_{m2} } & \cdots \\ \end{array} } & {\begin{array}{*{20}c} 1 & \cdots & {e_{\alpha m} } \\ \vdots & \ddots & \vdots \\ {e_{m\beta } } & \cdots & 1 \\ \end{array} } \\ \end{array} } \right]$$

The fusion of each Exhibit $U_{i}$(*i* = 1,2*,*$\ldots ,A$) yields an improved evidence fusion result of $X = (p_{x} \left( {Q_{1} } \right),(p_{x} \left( {Q_{2} } \right), \ldots ,(p_{x} \left( {Q_{M} } \right),\left( {p_{x} \left( \Theta \right)} \right)$. The improved decision criterion formula is from Eq. (11).11

Assuming there exists $Q_{g}$ and $Q_{h }$ if $Q_{g }$ satisfies the decision Eq. (12), the evaluation result is ${\text{X}}^{\prime }$. Otherwise, the decision is rejected. In Eq. (12), $\tau_{1} \in \left( {0,1} \right)$ and $\tau_{2} \in \left( {0,1} \right)$ are both thresholds set up for decision-making.

### Multi-view feature based integrated residual network

Combat images contain rich information dimensions, contributing to analyzing and evaluating combat effectiveness from different dimensions. It is possible to quickly provide feedback on the combat effectiveness of weapons and equipment during real-time processing of scene information by automatically recognizing images.

Image data corresponding to the indicators in the index system are extracted from three aspects: color space, shape texture, and visualization. The corresponding image multi-view features such as HSV, HI, and CIE view are generated. The multi-view features are used as input data for the improved residual CNN neural network to build the base classification models HSV_CNN, HI_CNN, and CIE_CNN respectively. The base classification models are integrated by different strategies, and metrics such as accuracy, precision, recall, and score are selected for evaluation.

**Figure 4:**
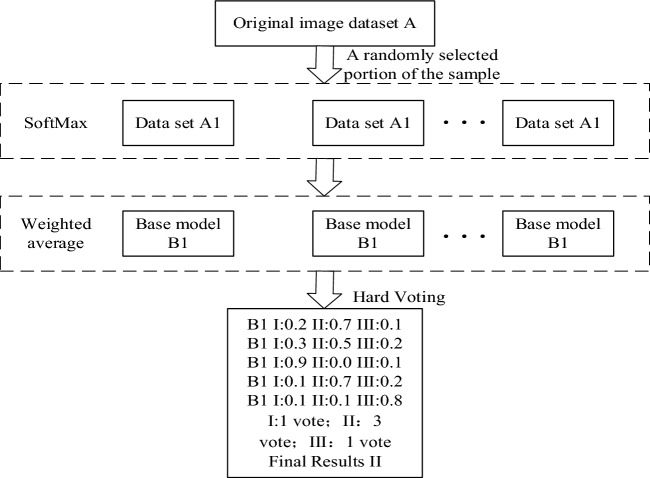
Flowchart of the bagging strategy module.

The Bagging algorithm randomly changes the training set distribution so that the new training subset is fused into the individual learner training to obtain the prediction results. Bagging sample sets are generated and each set is passed to the base model to select the SoftMax classifier to obtain the maximum probability distribution class. The output of the evaluation results is obtained after combining multiple models’ results for hard voting to construct a multi-view Bagging Integrated Network Model (B_CNN) module based on CNN networks. The flowchart is shown in Fig. 4.

**Figure 5:**
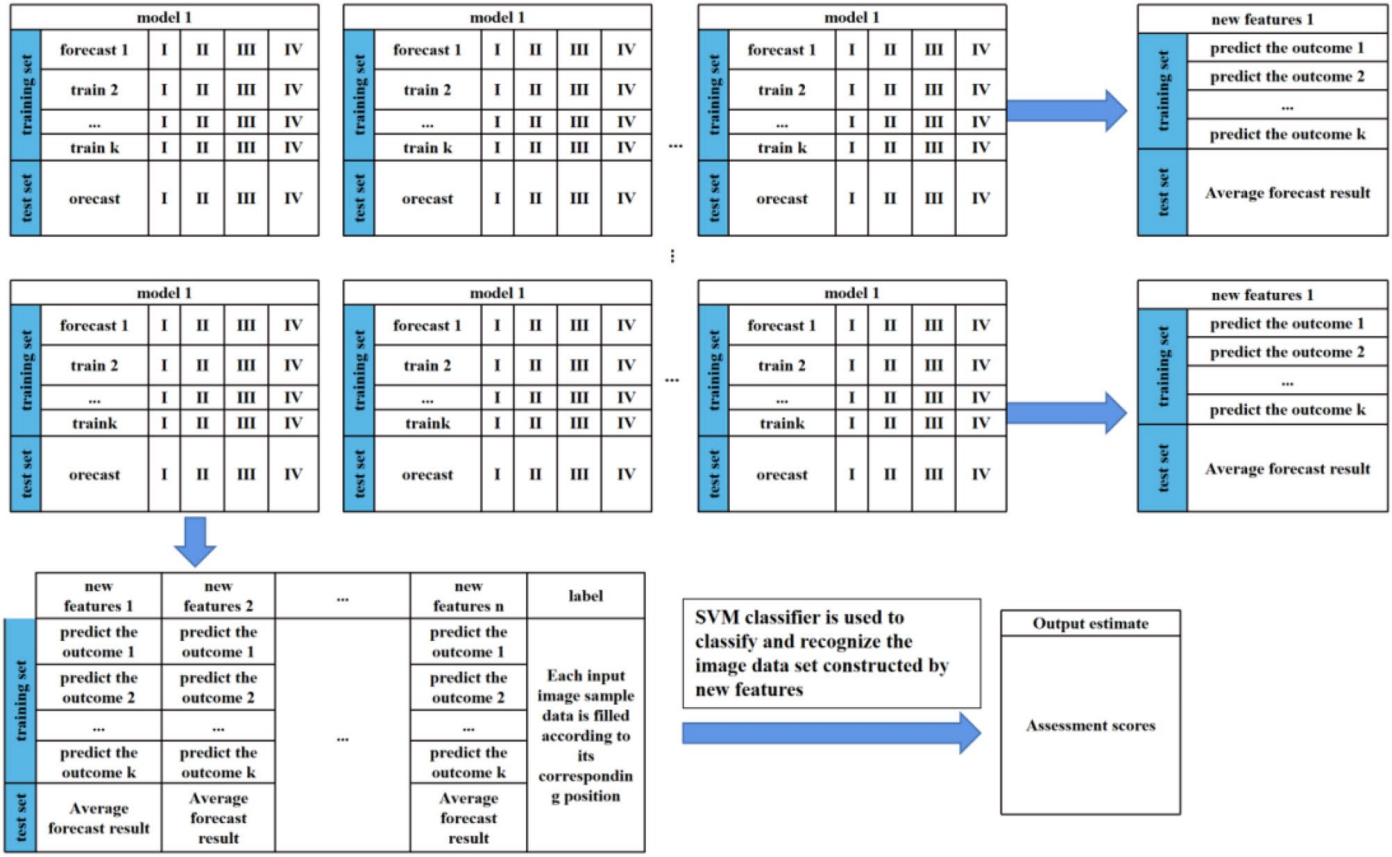
Stacking integration module flowchart.

The Stacking integration algorithm consists of a base classifier for data training and a meta-classifier for integrated data output. The data in the base classifier is crossed to produce four training set data and one test set data. The validated predictions are used as the training set for the meta-classifier model, and the predictions are averaged as the test set. The feature data of multiple models are compared with the sample labels to obtain the evaluation results. A multi-view Stacking Integrated Network Model (S_CNN) module based on the CNN network is constructed and SVM is used to classify and identify the fused data. The flow chart is shown in Fig. 5.

The multi-view features are cascaded with CNN networks to form a C_CNN module, which expands the data dimensionality to 500 dimensions per view and performs deep feature extraction to enhance the use of fuzzy image data with good generalization capability. After obtaining different view depth feature values and real label cascade, SVM is selected to classify and identify the deep multi-view feature data and output the evaluation results. A CNN-network-based depth extraction evaluation model (C_CNN) module is constructed and the flow of the algorithm is as follows:

**Figure:**
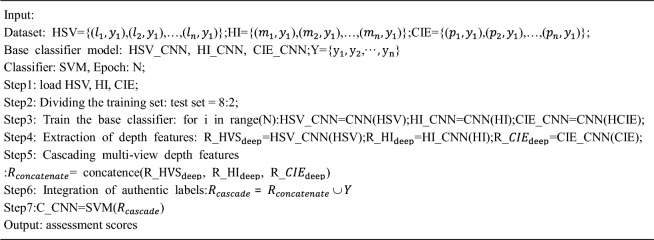
Algorithm: Multi view feature deep cascading network

**Figure 6:**
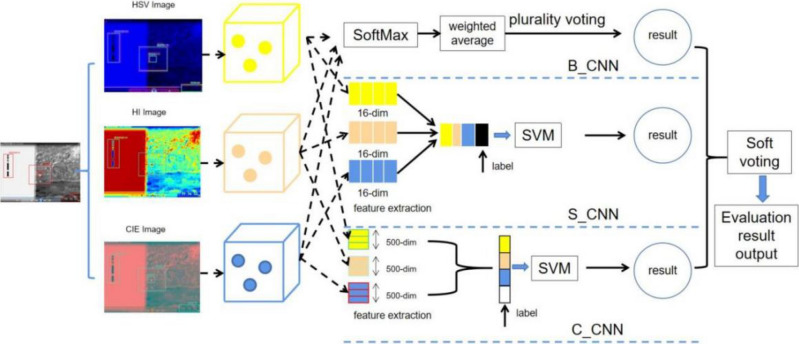
A framework for intelligent evaluation of multi-view feature integration networks.

**Figure 7:**
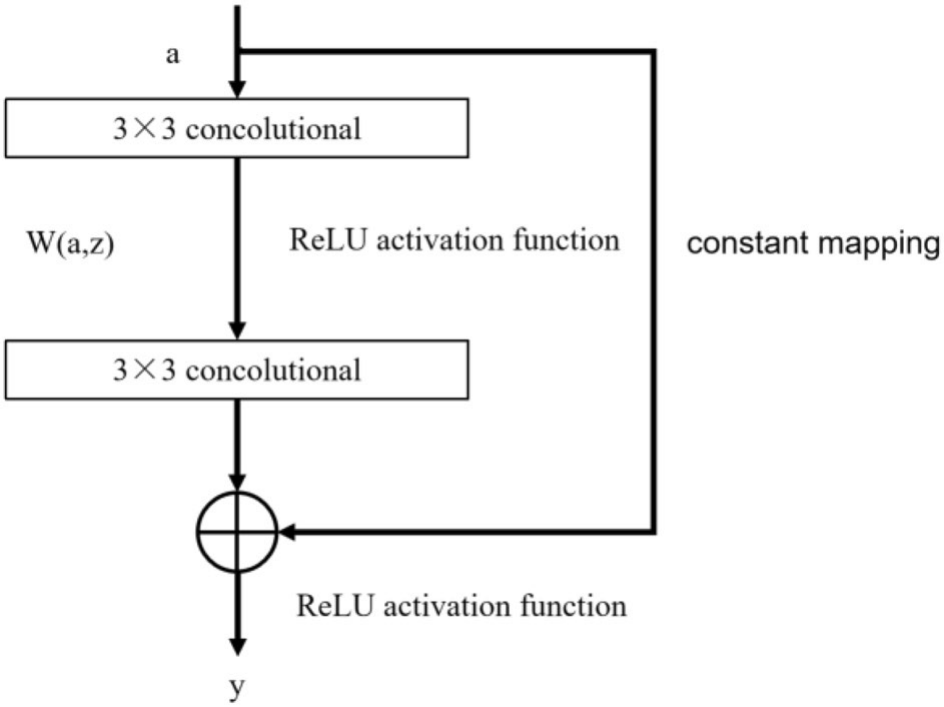
Improved residual network model.

The framework of the improved metric evaluation model for integrated multi-view learning is shown in Fig. 6. The evaluation results of the B_CNN, S_CNN, and C_CNN modules in the improved model are soft-voted, and the optimal evaluation scores are obtained by calculating the mean evaluation probabilities of different results. The improved assessment model can automatically extract feature data, fully fuse multiple types of data, and intelligently obtain an objective score without human interference. The improved model contains a total of 14 hidden layers, and the overall network structure is deeper. As shown in Fig. 7, the improved residual network structure is introduced to solve the problem of gradient disappearance and degradation in CNN network training and improve the indicator image classification recognition evaluation accuracy.

The output view feature value of the model is as Eq. (12).

12 $${\text{y}} = {\text{a}} + {\text{W}}\left( {{\text{a}},{\text{Z}}} \right)$$

In Eq. (12), *a* is the input view feature value of the model, *Z* is the input weight value, and *W*(*a,z*) is the residual mapping function. The input *a* is calculated after 2 layers of convolution and one activation of the residual mapping function, and then the constant mapping function input *a* is added to obtain the output *y* of the residual block. The parameters of each layer of the improved CNN neural network are shown in Table 1.

**Table 1:** Improved network parameters for each layer.

Number of network layers	Network type	Convolution kernel size/pixel	Output size/pixels
1	Convolutional layer 1	$7 \times 7$	$72 \times 72 \times 16$
2	Convolutional layer 2	$7 \times 7$	$66 \times 66 \times 32$
3	Convolutional layer 3	$7 \times 7$	$60 \times 60 \times 32$
4	Pooling layer 1	$2 \times 2$	$30 \times 30 \times 32$
5	Convolutional layer 4	$3 \times 3$	$28 \times 28 \times 128$
6	Convolutional layer 5	$3 \times 3$	$26 \times 26 \times 128$
7	Convolutional layer 6	$3 \times 3$	$24 \times 24 \times 128$
8	Pooling layer 2	$2 \times 2$	$12 \times 12 \times 128$
9	Residual blocks 1	$3 \times 3$ $3 \times 3$	$12 \times 12 \times 128$
10	Convolutional layer 7	$3 \times 3$	$10 \times 10 \times 256$
11	Convolutional layer 8	$3 \times 3$	$8 \times 8 \times 256$
12	Convolutional layer 9	$3 \times 3$	$6 \times 6 \times 256$
13	Pooling layer 3	$2 \times 2$	$3 \times 3 \times 256$
14	Residual blocks 2	$3 \times 3$ $3 \times 3$	$3 \times 3 \times 256$
15	Fully connected layer	$1 \times 1$	$1 \times 1 \times 2304$
16	Fully connected layer	$1 \times 1$	$1 \times 1 \times 1024$

## Experimental process and results

### Indicator system construction experiments and results

This article uses the YOLOv4 model for image recognition and uses the CSP-DarkNet53 network as the backbone network. To improve its detection range and accuracy, we adopted multi-scale feature extraction technology to capture image features at different levels. The learning rate of the main parameter is initially set to 0.00125, and continuously changing based on the cosine annealing algorithm, with a decay factor of 0.0001. In addition, the momentum coefficient is set to be 0.9 and the batch_size to be 2. After setting experimental parameters in the experimental scene, 300 iterations of training are conducted to recognize typical objects.

Take a video in the target search time of test scenario A as an example, the terrain in the video is mountainous, and the information in the lower right corner of the picture indicates that the center of the equipment’s white aiming frame is aligned with the equipment target at 19:34:55, in which the middle cross cursor is the target to be hit. The black stripe on the left side is due to shooting jitters.

**Figure 8:**
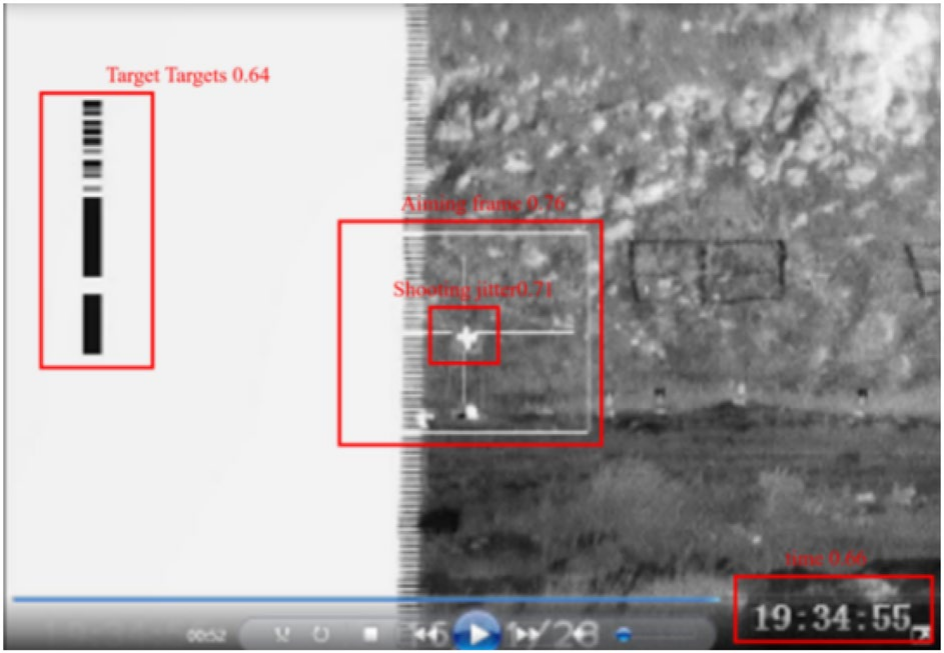
Test scenario A typical recognition object.

The typical object recognition in this scenario is shown in Fig. 8. The detection results for typical objects in ten test scenes are shown in Table 2. The detection result of “0” of a certain typical recognition object indicates that it is not detected in the scene, while “1” indicates its existence. The experimental results show that the average recognition accuracy of typical objects is 80.54%. The typical object with the highest recognition rate is the frequency hopping digit, which reaches 92.45%. The recognition accuracy of shooting time difference is only 31.82%, which is 48.72% lower than the average accuracy.

**Table 2:** Test scene recognition experimental results.

Typical identification objects	Testing test scenarios										Recognition rate(%)	Typical indicators
	A	B	C	D	E	F	G	H	I	J		
Aiming frame	1	0	0	0	0	1	1	0	0	0	90.08	√
Target Targets	1	0	0	0	0	1	1	0	0	0	91.04	√
Point in time	1	0	0	0	0	1	0	0	0	0	83.12	√
Shooting jitters	1	0	0	0	0	1	1	0	0	0	90.82	√
Dust disturbance	1	0	0	0	0	1	1	0	0	0	77.36	√
Targeting the dividing line	0	0	0	0	0	0	1	0	0	0	47.83	
Shooting time difference	0	0	0	0	0	1	0	0	0	0	31.82	
Number of ammunition	1	0	0	0	0	0	1	0	0	0	89.81	√
Target destruction	0	0	0	0	0	1	1	0	0	0	84.62	√
Indicator light on and off	0	1	0	0	1	0	0	0	0	0	90.95	√
Indicator colours	0	1	0	0	1	0	0	0	0	0	87.19	√
Three-proof alarm text	0	1	0	0	1	0	0	0	0	0	86.32	√
Switch status	0	1	0	0	1	0	0	1	0	0	89.09	√
Video time	0	0	0	0	1	0	0	0	0	0	25.64	
Engine load	0	1	0	0	0	0	0	1	0	0	87.91	√
Power parameters	0	1	0	0	0	0	0	0	0	0	35.64	
Instrument panel parameters	0	1	0	0	1	0	0	1	0	0	90.21	√
Driving parameters	0	0	0	0	0	0	0	1	0	0	22.45	
Vehicle nodes	0	0	1	1	0	0	0	0	1	0	87.31	√
Equipment status	0	0	1	0	0	0	0	0	1	1	92.21	√
System time	0	0	1	1	0	0	0	0	0	0	84.11	√
Fixed area text	0	0	1	1	0	0	0	0	0	0	77.78	√
Types of alarms	0	0	1	1	0	0	0	0	0	1	87.37	√
Map coordinates	0	0	1	1	0	0	0	0	1	1	92.28	√
Temperatures	0	0	0	0	0	0	0	0	0	0	33.08	
Wind speed	0	0	1	0	0	0	0	0	1	0	84.43	√
Light and dark	0	0	0	0	0	0	0	0	0	0	35.14	
Alarm threats	0	0	0	1	0	0	0	0	1	1	90.45	√
Frequency Hopping Digital	0	0	1	1	0	0	0	0	1	1	92.45	√

According to the research of Yang et al.^35^ and Zhu et al.^36^, the indicator’s recognition rate in images is used for indicator screening. Indicators with low recognition rates in images are difficult to obtain and train. In our research, indicators with a recognition rate of less than 50% were filtered out with 22 indicators retained. In addition, the information on excluded indicator items can be obtained by the calculation of retaining indicators. The excluded shooting time difference indicator item can be reflected by calculating the indicator item at the time point, and the power transmission parameters can be reflected through engine load, etc. Removing seven indicators may ensure the information integrity and indicator exclusivity of the final indicator system. The established combat effectiveness index system of a certain type of equipment based on image detection is shown in Table 3.

**Table 3:** Operational effectiveness index system.

System	Tier 1 indicators	Tier 2 indicators	Tier 3 indicators
A certain type of equipment combat effectiveness indicator system	Effectiveness of the use of firepower	Rapid response capability	Targeted search area
			Target recognition rate
			Target discovery time
		Cluster fire capability	Collected fire time
			Shooting effects
		High point destructive capability	Smoothness of ammunition supply
			Shooting accuracy
	Co-operative effect	In-vehicle collaboration capabilities	Battle preparation time
			Mission adaptability
		Equipment adaptability	Timeliness of communication
			Equipment failure rate
		Long-distance marching capability	Engine load
			Instrument panel parameters
	Command and control effects	Communication decision making capabilities	Network interoperability
			Information networking capabilities
		Situational awareness capabilities	Information mapping
			Posture reception success rate
			Posture update
		Ability to control movement	Location update correct rate
			Environmental adaptability
		Feature suppression capability	Anti-Laser Surveillance
			Active protection

### Indicator system assessment experiments and results

The different model intelligence evaluation experiments conducted for the established weaponry index system are all carried out on the same hardware device to ensure that the experimental results can be directly compared. The main parameters of the experimental platform are: CPU Intel Core i9-12900HK, graphics card RTX3050Ti, main frequency 5.6 GHz, running memory can be expanded to 128G, equipped with 64-bit Windows 10 system, programming environment for Matlab 2020a and Pytorch 1.8.0.

### Optimizing IPSO-BP neural network evaluation

The full weights and thresholds of the BP neural network are encoded, a group of particle swarms is randomly generated, and each particle in the swarm represents the full initial weight and threshold distribution of a neural network. A dimension in each particle represents a weight or threshold, and then the dimension of each particle is the number of all weights or thresholds of the neural network. Setting *m* as the number of neurons in the input layer,* n* as the number of neurons in the hidden layer, and* k* as the number of neurons in the output layer, from Eq. (13).

13 $${\text{l}} = {\text{mn}} + {\text{nk}} + {\text{k}} + {\text{n}}$$

The size of the particle population in the IPSO algorithm has an impact on the convergence speed and accuracy stability of the model. Combined with the number of indicators and the number of layers for constructing the indicator system, the number of nodes in each layer of the model neural network is set to be 22 nodes in the output input layer, 5 nodes in the implied layer and 1 node in the output layer. According to the calculation formula (13), the particle dimension and number are 121 and 85 respectively. Setting the maximum model iteration number as 300, the maximum and minimum values of inertia weights as 0.85 and 0.15 respectively, and the model learning rate as 0.001.

The error function of the IPSO-BP model is set to Eq. (14).

14 $${\text{e}} = \frac{1}{{\text{F}}}\mathop \sum \limits_{{{\text{n}} = 1}}^{{\text{I}}} \left( {{\text{y}}_{{\text{n}}} - \widehat{{{\text{y}}_{{\text{n}}} }}} \right)^{2}$$

$y_{n}$ and $\widehat{{y_{n} }}$ in Eq. (14) are the desired and actual scoring values of the IPSO-BP model respectively.

According to the processing results of the numerical value and the collected index item data information, statistics related to the index effectiveness index of the parameter effect data are generated to evaluate the experiment. The combat effectiveness index sampling data is shown in Table 4.

**Table 4:** Sample data on operational effectiveness indicators.

Type	Target discovery time	Firing effect coefficient	Instrument parameters	Environmental adaptation index	Active protection parameters	Shooting accuracy index	Battle preparation time	Timeliness of communications	Posture update parameters	Networking capacity factor
TX9	4.57	0.90	78.31	0.87	1.4	1.433	1.94	4.92	0.75	1.83
TX26	6.12	0.70	82.72	0.75	1.5	1.435	2.71	3.73	0.71	1.02
TX77	10.51	1.00	86.93	0.85	2.2	1.435	2.92	4.36	0.74	1.05
TX108	4.48	0.75	92.14	0.82	1.8	1.461	3.01	7.69	0.81	1.07
ZL11	3.09	0.80	66.45	0.73	0.8	1.788	2.72	6.58	0.92	1.05
ZL43	5.37	0.85	75.96	0.86	1.5	1.656	2.36	7.04	0.95	1.06
ZL140	6.53	0.85	81.37	0.92	3.1	1.794	2.48	5.36	0.93	1.03
ZL202	5.52	0.90	79.98	0.91	2.7	1.892	2.82	6.61	0.85	1.06

**Figure 9:**
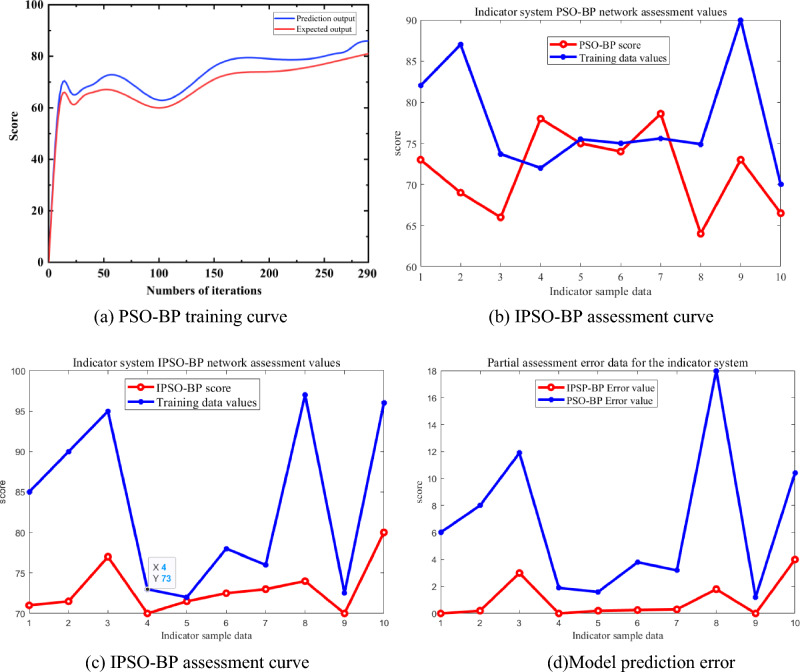
Comparison of model training, individual predictions and errors.

From Fig. 9a, it can be seen that the IPSO-BP network produces a decrease in prediction scores at the 30th and 100th generations, and both prediction scores and expectation scores rise gently after 100 generations. The overall curve of the improved model is flatter, with smaller prediction errors. From the random data sampling curves in Fig. 9b,c, the weights and thresholds of the optimized IPSO-BP network are constantly corrected by the cooperative and competitive optimization of the searching method between the particle swarms, so that its convergence speed is faster, and the searching efficiency and accuracy are effectively improved.

By setting the error function formula (14) and training to get the average error, the sampling data is calculated as shown in Fig. 9d. Each generation of the model will get the corresponding predicted value and the actual value, the predicted value of each generation will be subtracted from the average error and then the weighted average and the actual value to get the evaluation score of each generation of the indicator system. Through the evaluation score statistics of 300 generations, the optimal evaluation score of the indicator system constructed in this paper is 82.43 points.

### Improved DS evidence-parallel network evaluation

The evaluation index space $Z = \left\{ {Y_{1} ,\,Y_{2} , \ldots ,\,Y_{22} } \right\}$ is established, and the evaluation result identification framework is $\Theta$ = {$F_{1} ,\,F_{2} ,\,F_{3} ,\,F_{4} \,,\,F_{5}$}*.* The results of the system evaluation are divided into five levels ($W_{1}$*,* $W_{2}$, $W_{3}$*,* $W_{4} ,\,\,W_{5}$). The proposition is noted as $F_{i}$ (*i* = 1, 2, 3, 4,5) , indicating that the current level of the index system to be evaluated is $W_{i}$. The node number of the neural network’s input layer ${\text{B}}_{{\text{i}}}$(i = 1,2,…,9) is determined as 22 according to *Z* and $\Theta$. The nodes of the hidden layer are set as 6, 9, 10, 12, 14, 15, 17, 18 and 21. The number of nodes in the output layer is 5(${\text{C}}_{{{\text{n}}1}} {,}\,{\text{C}}_{{{\text{n}}2}}$,${\text{C}}_{{{\text{n}}3}}$, ${\text{C}}_{{{\text{n}}4}}$, ${\text{C}}_{{{\text{n}}5}}$). The neural network after completing the training the test sample set data are evaluated and the credibility of each neural network is calculated separately. The results of each part are shown in Table 5.

**Table 5:** Preliminary evaluation results for each neural network.

Neural networks	Output results $K_{i}$	Preliminary evaluation results $K_{i}^{ + }$	Threshold $\varepsilon_{i}$	Evaluation results $X^{^\prime}$	Credibility $\theta_{i}$
$B_{1}$	(− 0.2612, 1.1321, 0.0014, 0.0372, − 0.0142)	(0.0000, 0.6941, 0.2326, 0.0671, 0.0062)	0.2	$W_{2}$	0.8621
$B_{2}$	(0.6029, − 0.1985, 0.5238, 1.0241, 0.0248)	(0.2325, 0.2591, 0.0000, 0.3813, 0.1271)	0.2	$W_{3}$	0.6814
$B_{3}$	(− 0.216, − 0.3346, 0.7346, 0.3528, 0.3219)	(0.1966, 0.4651, 0.2934, 0.0000, 0.0449)	0.2	Refusal to give results	0.7235
$B_{4}$	(0.1499, − 0.0759, − 0.0182, 1.143, 0.0276)	(0.1994, 0.6490, 0.0233, 0.0000, 0.1283)	0.2	$W_{2}$	0.7463
$B_{5}$	(0.2247, 0.8361, − 0.6691, 0.7968, − 0.7629)	(0.2022, 0.3503, 0.1248, 0.3227, 0.0000)	0.2	$W_{4}$	0.4922
$B_{6}$	(− 0.3788, 0.9725, 0.0017, − 0.0218, 0.6555)	(0.0000, 0.4976, 0.1314, 0.0996, 0.2714)	0.2	$W_{2}$	0.8142
$B_{7}$	(0.2206, 1.1441, 0.1835, 0.2022, − 0.2481)	(0.1635, 0.5539, 0.1314, 0.1512, 0.0000)	0.2	$W_{3}$	0.6619
$B_{8}$	(0.1997, 0.9531, 0.0912, − 0.1915, − 0.3327)	(0.2217, 0.1414, 0.0000, 0.5576, 0.0793, )	0.2	$W_{2}$	0.7867
$B_{9}$	(0.4326, 0.0524, 0.1011, 0.9794, 0.0566)	(0.4514, 0.0000, 0.1126, 0.1315, 0.3045)	0.2	$W_{1}$	0.9021

As can be seen from Table 5, if only one BP neural network is used to evaluate the index system, the evaluation result can be determined directly from $B_{i}^{ + }$ as $X^{\prime}$. $B_{2}$ and $B_{7}$ give an evaluation grade of $w_{3}$ and $B_{3}$ refuses to give an evaluation result. The evaluation of each neural network is uneven with low confidence. Therefore, the data is fused using an improved DS evidence theory to reduce uncertainty and improve identification accuracy. Firstly, the output of the neural network $K_{i} \left( {i = 1,2, \cdots ,9} \right)$ is normalized. The confidence level $\theta_{i}$ is corrected to generate evidence $U_{i} { }$ to assign basic assignment probabilities to each proposition in the recognition framework, as shown in Table 6.

**Table 6:** Improved recognition results after data fusion.

Neural network	Credibility $\theta_{i}$	Evidence	($p_{i} (F_{1}$), $p_{i} (F_{2}$), $p_{i} (F_{3}$), $p_{i} (F_{4}$), $p_{i} (F_{5}$), $p_{i} ({\Theta }$)
$B_{1}$	0.8621	$U_{1}$	(0.0000, 0.6614, 0.1976, 0.0524, 0.0059, 0.0827)
$B_{2}$	0.6814	$U_{1}$	(0.1825, 0.1923, 0.0000, 0.3183, 0.0964, 0.2105)
$B_{3}$	0.7235	$U_{1}$	(0.1461, 0.4029, 0.2436, 0.0000, 0.0318, 0.1756)
$B_{4}$	0.7463	$U_{1}$	(0.1721, 0.4417, 0.0108, 0.0000, 0.0928, 0.2826)
$B_{5}$	0.4922	$U_{1}$	(0.1825, 0.2923, 0.1176, 0.2571, 0.0000, 0.1505)
$B_{6}$	0.8142	${\text{U}}_{1}$	(0.0000, 0.3761, 0.0814, 0.0569, 0.1781, 0.3075)
$B_{7}$	0.6619	$U_{1}$	(0.1112, 0.4009, 0.0926, 0.1080, 0.0000, 0.2873)
$B_{8}$	0.7867	$U_{1}$	(0.1722, 0.0990, 0.0000, 0.4343, 0.0618, 0.2327)
$B_{9}$	0.9021	$U_{1}$	(0.3841, 0.0000, 0.1015, 0.0913, 0.2614, 0.1617)

Data fusion of the above nine pieces of evidence using the improved DS evidence theory yielded the results: *F* = ($p_{i} (F_{1}$)*,*$p_{i} (F_{2}$)*,*$p_{i} (F_{3}$)*,*$p_{i} (F_{4}$)*,*$p_{i} (F_{5}$)*,*$p_{i} (\Theta$) = (0.0229,0.9618,0.0124,0.0019,0.0008, 0.0002). The thresholds in the decision quotient are all set to 0.2, resulting in a final output evaluation result of *X* = $W_{2}$ with a score of 86.16 points.

### Multi-view feature based integrated residual network evaluation

**Figure 10:**
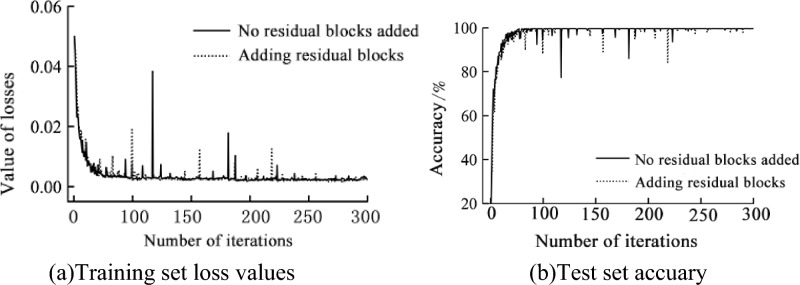
Changes in loss values and accuracy during training.

The number of iterations was set to 300 and the results of the training and test sets are shown in Fig. 10. As can be seen from Fig. 10a, the loss values in the training set are in a steep drop through the first 60 iterations, start to oscillate and converge by 80 iterations, and level off at 150 iterations. The model without the residual block spikes in loss around 120, 170, and 180 iterations and fluctuates unevenly. The model with the residual block added converges faster, with smoother fluctuations in the loss values at the later stages and better robustness and generalization. As can be seen from Fig. 10b, the model’s accuracy in the test set increases rapidly with the number of iterations, with the accuracy approaching 100% at 90 iterations. The overall structure is more stable with the addition of the residual block, with a maximum accuracy of 98.43%.

The experiments’ learning rate was set to 0.00001, the optimization function was the Adam algorithm, the Dropout value of the fully connected layer was set to 0.5, and the Epoch was 30. The SVM kernel function is a radial basis function with a penalty coefficient of 79 and a kernel function coefficient of 10.4. The training and test sets were divided into 8:2, and the input image size was 78 pixels × 78 pixels. The experimental results of each module constructed in the integrated evaluation model are obtained as shown in Table 7, and the score of the multi-view feature integrated residual network is 95.11 points.

**Table 7:** Results of different model scores.

Data	Model	Accuracy (%)	Precision (%)	Recall (%)	Score
Single-view	HSV_CNN	87.22	87.54	86.29	86.94
	HI_CNN	84.28	859	83.69	83.78
	CIE_CNN	79.94	80.11	80.22	80.03
Multi-view	B_CNN	88.54	89.27	89.02	88.91
	S_CNN	90.27	90.97	90.43	90.45
	C_CNN	92.45	93.17	93.04	92.86
	Framework	95.26	95.09	95.43	95.11

**Figure 11:**
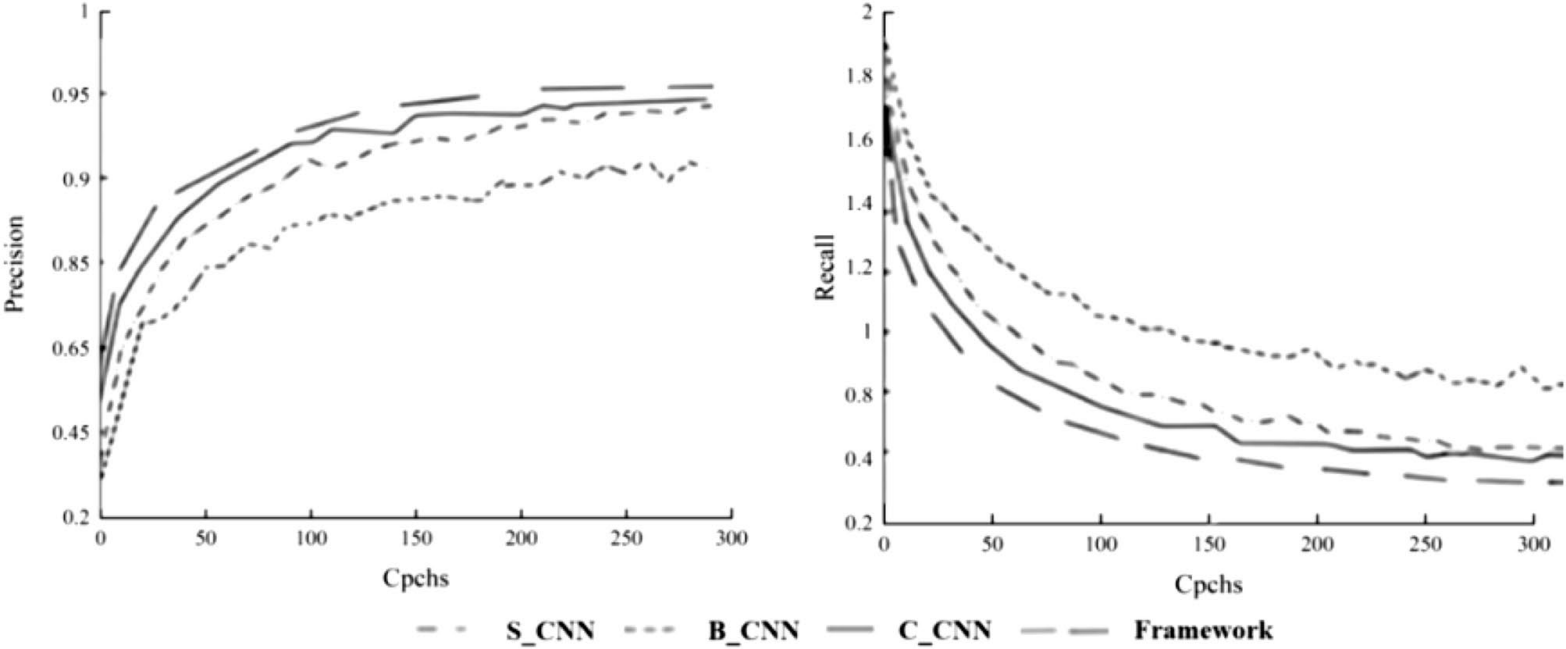
Precision training with loss function curves.

The recognition accuracy of the classification models HVS_CNN, HI_CNN, and CIE_CNN are 87.22%, 84.28%, and 79.94% respectively. The single view analysis shows that the extraction of color spatial features from image data is more effective than shape and visual features. The scores for evaluating the index system based on color spatial features are also 3.77% and 8.63% higher than those for evaluating shape and visual features respectively. The data of each index such as accuracy, recall, and score under multi-view are better than the single-view model. Its accuracy is 6.2%, 4.66%, and 2.25% higher than B_CNN, S_CNN, and C_CNN models. Its score is 6.2, 4.66, and 2.25 points higher than the B_CNN, S_CNN and C_CNN models respectively. The accuracy training and loss function variation of each recognition network is shown in Fig. 11. Each recognition network is stable during the validation process, but the integrated network shows significantly smoother and has better values.

## Analysis

Three algorithms are proposed in this paper: optimized IPSO-BP neural network, improved DS evidence-parallel network, and multi-view feature based integrated residual network. The three algorithms’ average evaluation score is 87.9 and the average running time is 34.17 s, all greatly improve the evaluation efficiency and accuracy while enriching the types of evaluation indexes. The comparison of results indicates the characteristics of the three intelligent algorithms. The optimized IPSO-BP neural network is the fastest, which is 3.89 s faster than the average, but has the lowest evaluation score, which is 5.47 points lower than the average score. The improved DS evidence-parallel network runs 5.64 s slower than the average time but has a higher evaluation score of 3.73 than the optimized IPSO-BP neural network. The situation where a single network produces a large error value is effectively solved in this algorithm. The multi-view feature based integrated residual network achieves the maximum score of 95.11, 15.38% higher than that of the optimized IPSO-BP neural network. Its running speed is 18.56% faster than that of the improved DS evidence-parallel neural network.

A total of six algorithms are selected for comparison analysis: subjective assessment of fuzzy hierarchical analysis and cloud model, objective assessment of SEM model and Bayesian network, and intelligent assessment of DNN neural network and Stacking integration. The comparison analysis results are shown in Table 8. The average evaluation score of the nine algorithms is 82.07 and the average running time is 34.23 s. The following results are drawn through Further analysis. (1) The evaluation scores of the nine algorithms evaluation algorithms are all above 75 points. According to the division of expert experience in subjective evaluation algorithms, above 75 points indicates excellent. It proves that the combat indicator system based on image recognition established in this paper is scientific and reasonable and has professional reliability. (2) The subjective assessment methods run faster. The fuzzy hierarchical analysis method is 15.99 s faster than the average time, but the assessment score is lower. The cloud model score is 6.88 points below the average score. (3) Objective assessment methods have higher assessment scores. The assessment score of the improved SEM model method is higher than the average score by 0.16, and the running time of the Bayesian network method is faster than the average time by 4.75 s. However, the computational volume is larger and the data model is not easy to build. (4) The DNN neural network and Stacking integration algorithm among the intelligent assessment algorithms both run at the slowest speed due to the model’s complexity. However, the assessment scores are higher than the subjective and objective methods, indicating that they need to be improved in running time and parameter selection. (5) The optimized IPSO-BP neural network algorithm ranks fourth among the nine algorithms in terms of both assessment score and running time. The evaluation score is higher than the subjective and objective evaluation algorithms, and the score is 9.62% higher than the subjective cloud model algorithm. The running time is 47.08% faster than the smart Stacking integration algorithm. (6) Improved DS evidence-parallel network ranked second among nine algorithms in terms of evaluation scores, with an improvement of 4.98% over the average score. In comparison with the same type of DNN neural network algorithm, running the network with 1 less layer and running 9.39% faster at the same time the evaluation score is 6.88 points higher. (7) Multi-view feature based integrated residual network evaluation score and running speed are better than the average score and average running speed of the nine algorithms, with an improvement of 15.89% and 5.58%, respectively.

**Table 8:** Comparison of the results of the various assessment methods.

Type	Evaluation algorithms	Description of features	Score	Speed (s)	vantage	drawbacks
Subjective assessment	Fuzzy hierarchical analysis^37^	Create a fuzzy analysis matrix based on the affiliation of indicators	76.02	18.24	The model can handle fuzzy indicator data, has a simple structure that is easy to adjust, and runs fast	Data acquisition and expert participation are demanding and can introduce subjective factors and errors
	Cloud Modeling^38^	Establishing five cloud models and three cloud digital features	75.19	21.37	Better able to deal with non-linear relationships between indicators and data that are difficult to quantify and describe	The method cannot be used if the problem cannot be modelled. High demands on model parameters, data, and algorithms
Objective assessment	SEM modelling^18^	22 underlying indicators and structural equation $\eta = B\eta + {\mathcal{F}}\eta +$ calculations	82.23	35.69	Complex systems are assessed well through the standardisation of indicators to analyse the relationship of different variables	Requires large sample sizes and specialised statistics, poorly explained causal relationships between indicators
	Bayesian Network^21^	Bayesian inference to determine the affiliation-probability transformation formula	78.96	29.48	Captures probabilistic relationships between indicator variables and graphically displays results based on prior probabilities	Inability to deal with dynamic variable relationships, high requirements for modelling and data volume
Intelligent Assessment	DNN Neural Network^23^	Independent factor m,number of model layers 10, epoch and batch_10 of 300 and 100 respectively	79.28	43.55	Handle high-dimensional data with strong feature extraction and learning capabilities	The model structure and parameters are numerous and dependent on initial parameter settings, with over-fitting problems
	IPSO-BP Network	See research method of this paper	82.43	30.28	With global and local search capability, it can solve local optimal problems with good convergence, stability and generality	The model will have a large error in the results of a particular generation of optimisation search, with a certain amount of randomness
	Improving DS-Parallel Networks	See research method of this paper	86.16	39.81	More data and parameters can be processed at the same time with high scalability	Higher requirements for computer hardware and more complex algorithm design
	Stacking integration^26^	Add new feature vector and perform PCA dimensionality reduction, learning rate 0.08, n_clusters = 3	83.29	57.22	Base models can be selected and combined according to the characteristics and needs of the problem, with high prediction accuracy	High model training complexity, poor interpretability and risk of over-fitting
	Multi-view feature integration	See research method of this paper	95.11	32.42	Provide more comprehensive information to characterise the data, with good model robustness, convergence and predictability	Integration of model features is difficult and computational complexity is high

In summary, the IPSO-BP neural network method has a faster running time and is suitable for real-time rapid assessment. However, the stability of the model operation is poor, and there will be large error scores during the operation, which requires high requirements for model training. The parallel structure in the improved DS evidence-parallel network method is more scalable and improves the speed of data processing. It is suitable for complex equipment and large data volume index evaluation, but the setting of different network parameters of the model is more cumbersome. The Multi-view feature based integrated residual network method directly converts image data into numerical values, automatically selects the optimal feature values in each view, and integrates the feature information of different views for comprehensive assessment. Though the algorithm is complicated to test when selecting view feature types and setting integrated model parameters, it performs the highest score, good model generalization, and robustness while enriching the types of weapon and equipment evaluation indexes.

## Conclusion

This paper advances the method for constructing the combat effectiveness indicator system of weaponry based on image recognition for the first time and further proposes different intelligent assessment algorithms. On the one hand, by combining key combat effectiveness indicators with ten specific combat scenarios, a combat effectiveness indicator system consisting of 22 image indicators is proposed. On the other hand, three intelligent assessment methods, namely, optimized IPSO-BP network, improved DS evidence-parallel neural network, and Multi-view feature based integrated residual network, are proposed for the index system assessment. Experimental results show that all three improved assessment methods can realize the full intelligent assessment process from indicator data input to result output. Among them, the IPSO-BP network model belongs to the single network optimization assessment method and has the highest intelligent assessment efficiency. In addition, the method has lower performance requirements for the operation platform and can be widely used in portable platforms for field operations. The improved DS evidence-parallel neural network algorithm sets multiple neural networks, which can effectively reduce the interference of outliers in the evaluation of indicators. The adaptability of the model can be improved by adjusting the parameters of each neural network. However, it takes a long time to evaluate. The multi-view feature based integrated residual network model realizes the evaluation process from image input to result output. The accuracy and recall of this method are above 95%, and the model intelligent evaluation is optimal.

There are three main limitations to this study. Firstly, the experimental results of the IPSO-BP model are greatly influenced by the quality and quantity of input data, and the application of this method has high requirements for data acquisition and processing. Secondly, running the DS evidence-parallel neural network model in an environment with limited computing resources may be a challenge. Other models and methods can be considered in the future, and compatibility between models can be adjusted to further optimize the intelligent evaluation method of the indicator system.

## Data Availability

All data generated or analyzed during this study are included in this published article, the corresponding author would like to provide more data on reasonable request.
